# Multi-scale detection of pulmonary nodules by integrating attention mechanism

**DOI:** 10.1038/s41598-023-32312-1

**Published:** 2023-04-04

**Authors:** Zhenguan Cao, Rui Li, Xun Yang, Liao Fang, Zhuoqin Li, Jinbiao Li

**Affiliations:** School of Electrical and Information Engineering, Anhui University of Science and Technology, Huainan, 232001 Anhui China

**Keywords:** Lung cancer, Oncology

## Abstract

The detection of pulmonary nodules has a low accuracy due to the various shapes and sizes of pulmonary nodules. In this paper, a multi-scale detection network for pulmonary nodules based on the attention mechanism is proposed to accurately predict pulmonary nodules. During data processing, the pseudo-color processing strategy is designed to enhance the gray image and introduce more contextual semantic information. In the feature extraction network section, this paper designs a basic module of ResSCBlock integrating attention mechanism for feature extraction. At the same time, the feature pyramid structure is used for feature fusion in the network, and the problem of the detection of small-size nodules which are easily lost is solved by multi-scale prediction method. The proposed method is tested on the LUNA16 data set, with an 83% mAP value. Compared with other detection networks, the proposed method achieves an improvement in detecting pulmonary nodules.

## Introduction

Lung cancer^1,2^ is a malignant tumor with the highest morbidity and mortality. If it can be detected and treated early, the survival rate of patients will be greatly improved^3^. As one of the important clinical manifestations of lung cancer in the early stage, accurate identification and localization of pulmonary nodules are of great significance. According to the “China Artificial Intelligence White Paper”, each patient will generate about 200 to 300 CT images during the examination process, and each radiologist will read at least 40,000 images per day. Unlike machines, the energy and accuracy of doctors will decline after long hours of mechanical reading every day, which may lead to missed diagnoses^4^. At the same time, the 30% increase in imaging examination growth and the 4% increase in imaging doctor resources each year have given computer-aided diagnosis (CAD)^5^ a broad development prospect. CAD technology can not only reduce the workload of doctors but also provide reliable reference information. At present, many researchers have proposed various effective detection algorithms^6,7^. Including traditional machine learning methods, morphological-based analysis, using threshold segmentation, dilation, erosion, and other operations to extract characteristic information of pulmonary nodules, and then using classification algorithms to detect pulmonary nodules. Sameh et al.^8^ used the Otsu threshold to segment lung nodules, then used morphological operations to extract features such as geometry and texture, and fused the selected best features to classify lung nodules. Saba et al.^9^ proposed an automatic classification and detection method, including lesion enhancement, segmentation, and feature extraction, and finally used a multi-classifier for classification and regression to achieve the detection of lung nodules. This method relies on manual screening. For different types and sizes of pulmonary nodules, the generalization ability is not strong, and the expression ability of characteristic information is also limited.

Deep learning algorithm is a field of machine learning, which automatically recognizes features through a neural network and has a stronger representation ability for complex feature information. With the continuous development of deep learning, it has shown excellent performance in the application direction of concentrated computer vision, inspiring some researchers to apply object detection to the field of lung nodule detection. For example, Ying et al.^10^ applied the RCNN series of target detection algorithms to the detection of pulmonary nodules, and compared and analyzed the detection effects of the RCNN, Fast-RCNN, and Faster-RCNN algorithms, and analyzed some parameters (such as Dropout, Batch Size, etc.) on the accuracy. Some researchers have also designed different networks for the characteristics of lung nodules to achieve better detection results. For example, Cao et al.^11^ constructed a two-stage target detection network based on the Faster-RCNN framework. The first stage is used to detect as many candidate lung nodules as possible, and the second stage is used to reduce false positives of lung nodules. Wang et al.^12^ performed transfer learning on the edge detection network and improved the model into a multi-resolution lung nodule detection model suitable for image classification. Li et al.^13^ improved the Mask-RCNN network by exploiting the dense block structure of densenet and the convolution method of channel shutt. To obtain improved detection, researchers tried to obtain more spatial information to improve the detection effect, Ning et al.^14^ designed a 3D residual network (3D-ResNet) based pulmonary nodule auxiliary diagnosis system, which sliced along three different axial planes. The information is fed into the constructed network, and joint decision-making generates detection results. Different from the traditional slices that use x, y, z planes, Zheng et al.^15^ use a combination of different planes for training. These designs all reflect the researchers’ attempt to improve the detection effect by mining more dimensional image information^16^.

In the detection of lung nodules using deep learning methods, the following issues need to be considered^17^:

1. Due to different densities^18^, pulmonary nodules can be divided into solid pulmonary nodules and subsolid pulmonary nodules, and the morphological characteristics of different types of pulmonary nodules are different.
2. The size of pulmonary nodules ranges from 3 to 40mm^19,20^, and small pulmonary nodules are easily submerged during training, resulting in missed detection.

To solve the problems in lung nodule detection mentioned above, this paper designs a multi-scale lung nodule detection network based on the attention mechanism. In the data preprocessing stage, this paper uses a pseudo-color processing method to introduce contextual information, thereby enhancing the emphasis on the texture features of lung nodules. In the feature extraction part, the improved ResSCNet network is used, and the feature pyramid is used to fuse multi-scale features, so as to realize the detection of lung nodules with different traits and sizes. The contributions of the study are summarized as follows.

1. A pseudo-color processing strategy is designed to enhance the texture information of pulmonary nodules and include more semantic and Spatial information around pulmonary nodules.
2. Based on the attention mechanism, the ResSCBlock module is designed, and a feature extraction network is built based on this module. At the same time, a feature pyramid is introduced for feature fusion, which enhances the prediction ability of small-sized nodules, and generally improves the network's ability to predict multi-sized pulmonary nodules. predictive ability.

The structure of this paper is as follows: “Methods” section presents the specific information of the proposed network, “Experiment” section presents the experimental setup and the dataset used, and "Results and analysis" section presents the experimental results and their related analyses.

## Methods

### A multi-scale pulmonary nodule detection network fused with attention mechanism

The detection task of lung nodules can be performed as an object detection task in deep learning. The image X is input into the trained network, and the coordinate information [x, y, w, h] of the lung nodule and the corresponding probability P of the lung nodule are output. In view of the problems in the detection of pulmonary nodules mentioned above, this paper proposes a multi-scale pulmonary nodule detection network based on the attention mechanism to achieve better detection results. The model used is similar to the Faster-RCNN network model. The feature extraction part of the original network uses ResNet for feature extraction, and a skip link is introduced to directly lead the input to the output. The structure of this residual link can improve the gradient disappearance or gradient explosion issues caused by the deep network level for neural networks and has achieved good detection results in practical applications.

**Figure 1:**
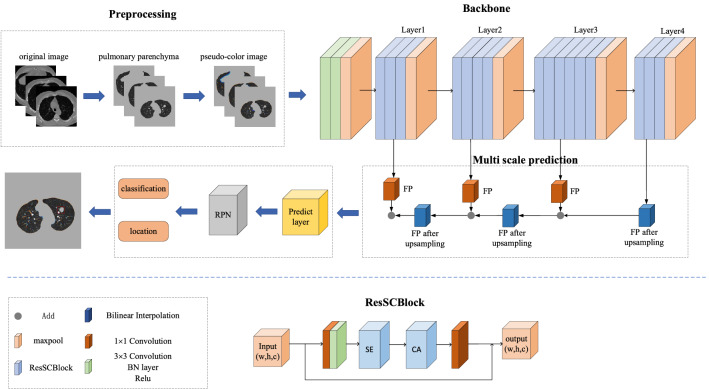
The constructed multi-scale pulmonary nodule detection network integrating attention mechanism is shown on the top, and the constructed basic module ResSCBlock is shown on the bottom.

The lung nodule detection network designed in this paper consists of four parts: data preprocessing, feature extraction, feature fusion part and region proposal network, as shown in Fig. 1.

In data preprocessing, the original lung CT image is segmented by lung parenchyma, and the image obtained by pseudo-color processing is used as the network input. Considering the following factors, the image is not cropped in this paper: 1. The position of the lung nodule relative to the lung is one of the spatial features of the lung nodule^21^. 2. The entire CT image contains more and more complex information, which increases the difficulty of detecting pulmonary nodules. On the other hand, it verifies the network’s ability to detect complex pulmonary nodules.

This paper proposes the basic module of ResSCBlock and builds a feature extraction network based on this module, which includes five stages. The backbone network consists of the improved ResSCNet network and the region proposal network. The improved ResSCNet network is composed of the basic module ResSCBlock proposed in this paper, which retains the original residual network link in the module, and introduces a compression excitation mechanism and a coordinate attention mechanism for feature fusion. The first stage includes 3 × 3 convolutional layers and maxpool layers, and 3 × 3 convolutional layers including the BN layer and Relu activation function. The second to fifth stages consist of several ResSCBlock basic modules and maxpool layers, the specific numbers are shown in Fig. 1. The ResSCBlock module extracts features, only changes the number of channels, but does not change the size of the feature map. The maxpool layer reduces the size of the feature map without changing the number of channels.

With the deepening of the network, the size of the feature map decreases in size, and the information contained increases in abstraction^22^. The deep feature map contains rich semantic information, but the location information is destroyed. The shallow feature map contains less abstract information, but the location information is more accurate. By using feature pyramids, predictions are made on multiple feature maps, ensuring that small-sized lung nodules are not missed. This paper uses the feature maps obtained after the second to fifth stages of processing. After fusion, the region proposal network is used for location prediction, and after non-maximum suppression (NMS), the final prediction is performed to obtain coordinate information and probability values.

### ResSCBlock

The designed ResSCBlock module introduces the compression excitation mechanism and coordinate attention mechanism based on the ResNet Block. The structure is shown in Fig. 1. This module extracts features from the input feature map X using 1 × 1, 3 × 3 and 1 × 1 convolution, and each layer of convolution is followed by a BN layer and a Relu activation function. The feature maps are then sequentially fed into the compressed excitation unit and the coordinate attention unit.

**Figure 2:**
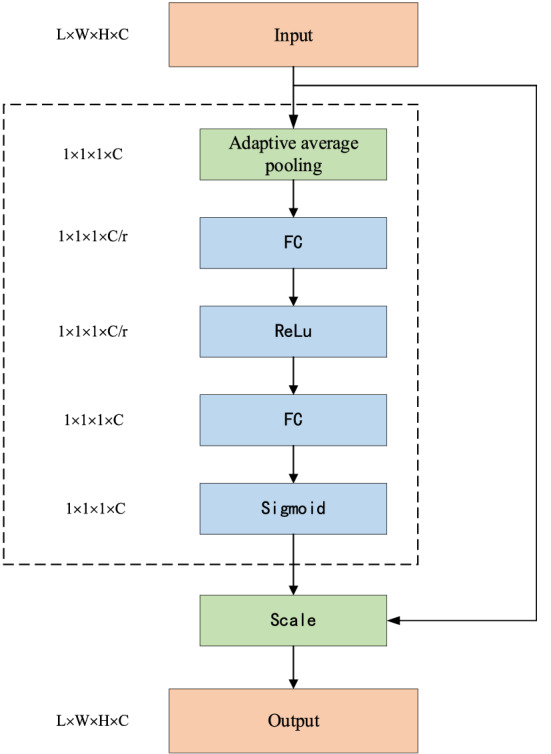
SE module structure diagram.

Squeeze excitation unit^23^ (Squeeze-and-Excitation model, SE), the structure is shown in Fig. 2, the unit includes two steps of compression and excitation. Assuming that the size of the input feature map X is [w, h, c], during compression, after the global average pooling operation, the size of the feature map obtained is 1 × 1 × C. The purpose of this operation is to fuse local features to obtain global features by using a pixel to replace the entire image of the channel. In excitation, after two fully connected layers, each fully connected layer contains an activation function. The number of neurons in the first layer of full connection is C × SERatio, SERatio is the activation parameter, the second layer contains C neurons, and the output size is 1 × 1 × C. Finally, after adjustment by the Scale operation, the weight of the output is multiplied by the input to adjust the weight of each channel of the input. Decrease the weight of the channel that does not contain the target, and increase the weight of the channel that contains the target.

Although the compressed excitation module reassigns the weights to each channel, it does not deal with the position information effectively. The commonly used bottleneck attention module (Bottleneck Attention Module, BAM) and convolutional block attention module^24^ (Convolutional Block Attention Module, CBAM) try to perform global pooling operation on the channel to introduce coordinate information, but receive local information, being unable to get information on long-range dependencies. The coordinate attention mechanism considers the relationship between channels and position information at the same time, so that the model can locate the target area more accurately.

**Figure 3:**
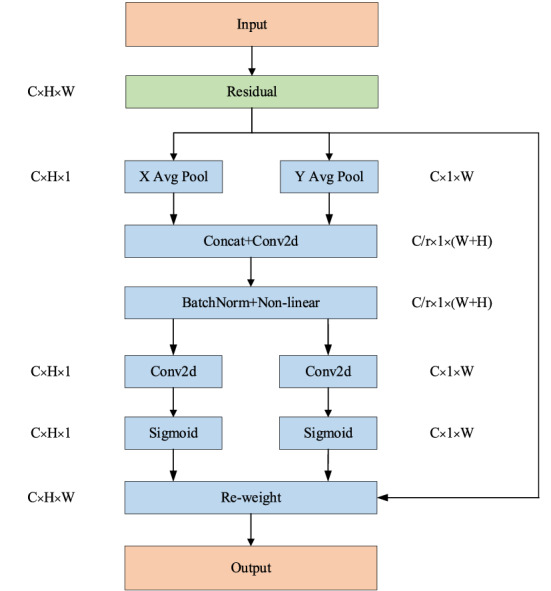
Coord module structure diagram.

The coordinate attention mechanism^25,26^ (Coordinate Attention, hereinafter the module is denoted as Coord) is shown in Fig. 3, including two parts: information embedding and attention generation. In the information embedding part, average pooling is performed in the horizontal and vertical directions to obtain two one-dimensional vectors. The attention generation part is spliced ​​in the spatial dimension, and the channel is compressed by 1 × 1 convolution. After the spatial information is encoded by the transform, the spatial information is weighted and fused in the channel dimension.

### Attention mechanism position

This section mainly discusses how adding an attention mechanism can achieve the best results. We first try to add the attention module into the network as a layer of the network. Scheme C1 is to add an attention mechanism module after each feature extraction layer, and perform feature fusion for shallow to deep features. Solution C2 is to add a feature fusion layer composed of three attention modules after the fourth layer to enhance the same extracted features. The above two schemes are to perform feature fusion on the features extracted by the residual layer through the attention mechanism. Next, try to combine the attention mechanism with the residual module, respectively in 1*1, 3*3, 1*1 Add attention mechanism modules after convolution and skip links, that is, C3, C4, C5, and C6 schemes. These schemes enhance features immediately after feature extraction. According to the experimental results, the scheme that can obtain the best effect is determined. The detailed experimental results are shown in Table 1.

**Table 1:** Results of using attention mechanism with different numbers of such layers in different locations in the network.

Attention mechanism position	mAP
C1(layer1-4*1)	0.7584
C2(layer4*3)	0.7786
C3(Block-1*1)	0.7467
C4(Block-2*1)	0.8308
C5(Block-3*1)	0.7600
C6(Block-Add*1)	0.5709

### Fusion multi-scale feature prediction

With the deepening of the network, the extracted features contain more abstract and richer information. Then comes the problem of feature information level and training level. At the feature level, in the shallow part of the network, the feature information is more specific and contains more location information, and the features at this level are more advantageous for small-size target prediction. With various convolution pooling operations, the features become increasingly abstract, and the location information is also removed. The feature information of small-sized pulmonary nodules is likely to be submerged, but the features containing more semantic information include the more information there is, the more advantageous it is for the detection of large-size targets. To address this problem, this paper introduces feature pyramids.

The feature maps extracted from the second to fifth layers are input into the feature pyramid for feature fusion. The specific operation is to first use 1 × 1 convolution to adjust the number of channels to 256 for the top-level feature map, and then adjust the size to the previous one through up sampling. The layer feature maps are consistent, denoted as L5. The up sampling operation selected in this paper is bilinear interpolation, and the channel number selection 256 is determined according to the channel number of the L2 layer feature map. The second step is to keep the size of the front layer feature map through 1 × 1 convolution, and adjust the number of channels to 256. Then the two adjusted feature maps are added together for fusion. The above operations are performed on the fused feature maps in turn with the previous layer feature maps, and finally three fused feature maps L4–L2 are obtained. In addition, the top-level feature map is max-pooled to obtain L6. Finally, the region proposal network is used to make predictions on L2–L6. After the NMS operation, the final prediction is made, the classification confidence P, and the predicted lung nodule location [x, y, w, h] are given.

### Pseudo-color processing strategy

The data set in this paper is produced through the consideration of the need to predict the entire image in actual use, such that the original image size is retained. The Z-dimension image corresponding to the center of the nodule and four adjacent images were selected for pseudo-color processing, that is, three grayscale images were used as RGB three channels to synthesize a pseudo-color image. After this processing, it can be seen that the direction information of the processed nodules is reflected in the detail part, so that the image contains more contextual information.

**Figure 4:**
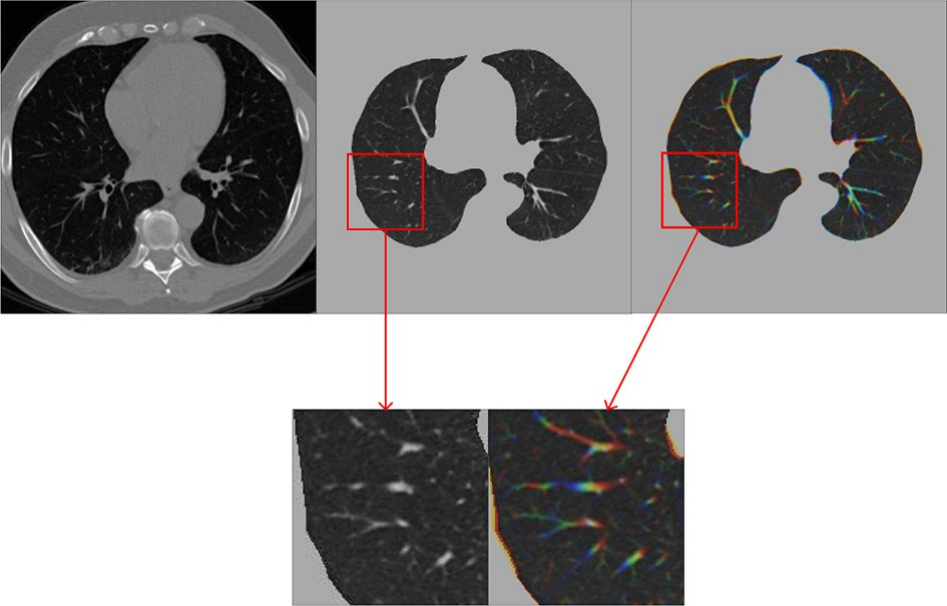
The one on the left of the first row represents the original image, the middle image of the first row is obtained after lung parenchyma segmentation, and the right image of the first row is obtained after pseudo-color processing. The second row shows the enhancement of texture features after pseudo-color processing.

The specific operation process is as follows^27^:

1. Normalized: Different scanners have different resolutions, so normalize the CT image data to 1 mm × 1 mm × 1 mm to eliminate the differences^28^.
2. Segmentation is performed according to the HU value: The HU value of human tissues varies, among which the HU value of water is 0, and the HU value of other tissues can be calculated according to the formula. Since the nodules were only present in the lung parenchyma, other tissues were removed by threshold segmentation.
3. Lung parenchyma was segmented: Lung parenchyma is segmented through operations such as inflation and inversion.
4. Three images before and after are selected, and pseudo-color images will be obtained according to the pseudo-color processing method, and the results are shown in Fig. 4.

### Loss function

Since this paper regards lung nodule prediction as a target detection task, the loss function consists of two parts, one is the classification error of whether the predicted area is a lung nodule, and the other is the regression error of the offset between the predicted area and the real area.

1 $$\it \it {\text{loss}} = {\text{loss}}_{{{\text{cls}}}} \left( {{\text{p}},{\text{m}}} \right) + {\upalpha }\left[ {{\upomega } \ge 1} \right]{\text{loss}}_{loc} \left( {t^{m} ,\nu } \right)$$

$P$ is the softmax probability distribution predicted by the classifier;  $M$ corresponds to the target ground-truth class label.

The classification error uses softmax multi-class cross-entropy loss:

2 $$\it \it {\text{loss}}_{{{\text{cls}}}} = - \sum\limits_{i = 1}^{n} {m_{i}^{*} \log {\text{m}}_{i} }$$

$m_{i}^{*}$ is the real label value; ${\text{m}}_{i}$ is the confidence that the classifier predicts that the current frame is category i;

The boundary regression loss adopts the Smooth L1 loss function.

3 $$\it {\text{loss}}_{{{\text{loc}}}} = \sum\limits_{i = 1}^{n} {smooth_{l1} (t_{i}^{m} -\upnu _{i} )}$$

## Experiment

### Data set

**Figure 5:**
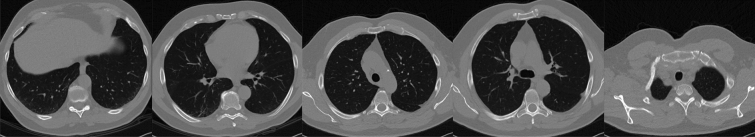
LUNA16.

This paper uses the LUNA16^29,30^ (Lung Nodule Analysis 2016) dataset for experiments. Luna16 comes from LIDC-IDRI^31,32^ (lung image database consortium and image database resource initiative), the largest public lung CT image database. After screening CT images with slice thickness greater than 2.5 mm, 888 CT images remain, with slice thickness ranging from 0.6 to 2.5 mm, with a spatial resolution of 0.46–0.98 mm and an average diameter of 8.3 mm. The criterion for nodules in the LUNA16 dataset was that at least 3 of the 4 radiologists considered the nodules to be larger than 3 mm in diameter, and a total of 1186 positive nodules were annotated in this dataset. LUNA16 data is in the mhd format (image information is stored in mhd files, and pixel information is stored in raw files). Each piece of data is a CT sequence, which contains three dimensions: each image is the X, Y dimension, and the sequence is the Z dimension^33^. A single CT sequence may contain multiple lung nodules at different locations. The dataset is first divided, and then data augmentation is performed according to the above method. LUNA16 is shown in Fig. 5.

### Experimental setup

The operating system used in the experiment is on a Windows 11 system, using an Intel(R) Core (TM) i5-12600KF CPU with a main frequency of 3.69 GHz, and a memory of 16 GB. The network model is based on the Pytorch deep learning framework and built with Python for version 3.7. Experiments were performed on an NVIDIA GeForce RTX 2060 graphics card with 12 GB of video memory. The data set has a total of 3558 pictures, and the training set and verification set are obtained after random division. The training set includes 3202 pictures, and the verification set includes 357 pictures. The optimization method used for network training is Stochastic Gradient Descent (SGD). The initial learning rate is 0.01, the weight decay coefficient is 1*104, the batch size is set to 8, and each experiment is trained for 50 rounds. The basic algorithm of the network selects the Faster-RCNN algorithm, and the training strategy of the network selects the joint training method of RPN loss and Faster R-CNN.

### Evaluation indicators

For the target detection task carried out in this paper, the evaluation index selects Mean Average Precision (mAP)^34^. At the same time, in practical applications, the missed detection of lung nodules is a more serious situation, so this paper chooses the recall rate to evaluate the detection performance of the network. Calculated as follows:

Recall is used to represent the proportion of positive samples that are correctly predicted to all positive samples. Calculated as follows:

4 $$\it {\text{Re}} {\text{call}} = \frac{TP}{{TP + FN}}$$

Precision represents the proportion of correctly predicted positive samples to all predicted positive samples. The formula is as follows:

5 $$\it \ {\text{Precision = }}\frac{TP}{{TP + FP}}$$

The area value of the PR curve drawn according to the precision rate, the recall, and the coordinate axis is the AP value, Only one category is set, so the AP value is equal to the mAP value:

6 $$\it AP = \frac{{\sum\limits_{i = 1}^{n} P }}{N(Totallmages)}$$

## Results and analysis

**Figure 6:**
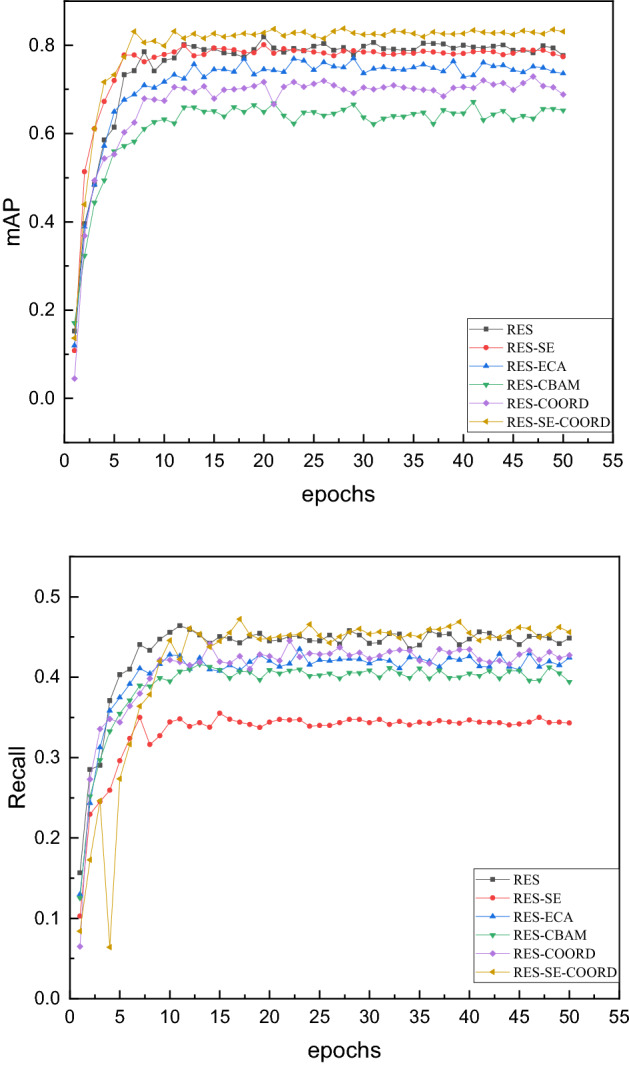
The yellow curve is the method used in this paper.

**Figure 7:**
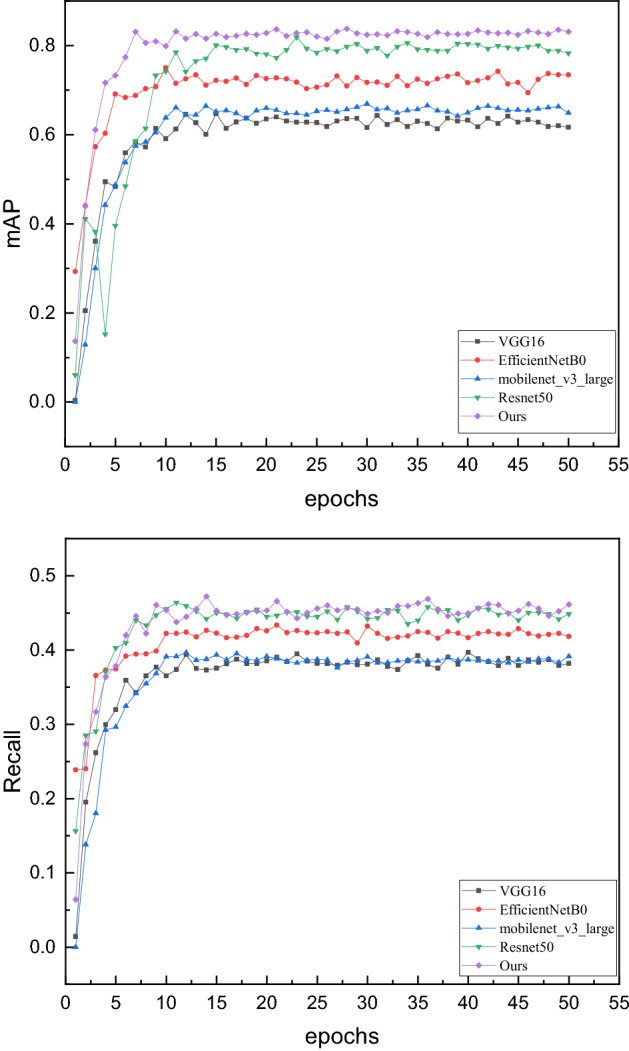
The purple curve is the method used in this article.

For the lung nodule detection network proposed in this paper, three groups of experiments are designed to verify its effectiveness. The first group uses ResNet50 as the basic module to compare different attention mechanisms. The results are shown in Table 2, and the change trend is shown in Fig. 6. The second group is compared with the mainstream detection network backbones, the results are shown in Table 3, and the change trend is shown in Fig. 7. The third group is compared with the mainstream target detection algorithm. the results are shown in Table 4.

**Table 2:** Comparison of detection effects of different attention mechanisms.

Method	mAP	Recall
Res	0.7915	0.4522
Res-SE	0.7735	0.4635
Res-Coord	0.7289	0.4314
Res-ECA	0.7402	0.4241
Res-CBAM	0.6504	0.4117
Ours	0.8308	0.4565

**Table 3:** Comparison of detection accuracy of different networks.

Method	mAP	Recall
ResNet50	0.7915	0.4522
Vgg16	0.6164	0.3820
Efficientnet-B0	0.7342	0.4221
mobilenet_v3_large	0.6487	0.3913
Ours	0.8308	0.4565

**Table 4:** Model comparison.

Method	mAP	Recall
SSD	0.6579	0.3181
Retainnet	0.8205	0.3936
Faster-Rcnn	0.7915	0.4522
Ours	0.8308	0.4565

Experiments1, 2, 3, and 6 are ablation experiments. According to the experimental results, the detection effect of using the SE module or the COORD module alone is worse than that of the original network, while using the ResSCBlock module proposed in this paper, both evaluation indicators have been improved.

This paper integrates the compression incentive mechanism and the coordinate attention mechanism. In order to verify the detection effect, this paper designs the above 6 sets of experiments (all using FPN). The experimental results show that the model tends to be stable around the 25th epochs. The network performance of this paper is the most stable and has strong robustness. Throughout the training process, our network consistently outperformed other networks. It shows that the fusion of the attention mechanism can improve the detection of pulmonary nodules. Compared with other schemes, the method in this paper improves by least 3.7% and 0.95%, and can give an improvement of up to 26.2% and 10.8%. Among them, the Res-SE scheme is 1.5% better than the scheme in this paper in the Recall index.

In this paper, the SE^35^ module and the Coord module are combined, and compared with other attention mechanisms, the optimal detection effect is obtained. At the same time, it shows that the lung nodule detection network proposed in this paper has a better detection effect. Therefore, for the detection of pulmonary nodules, the multi-dimensional enhancement of the channel dimension and the spatial dimension can improve the detection effect of pulmonary nodules.

Among them, the SE module fuses local information into global information through compression operation, and assigns high weight to target information by assigning reduced weight to unimportant information in the channel dimension. Therefore, using the SE module alone can improve the detection ability of nodules, but the classification ability of easy-to-mix structures is still subpar. From the experimental results, the performance of ECA (Efficient Channel Attention, ECA) is slightly worse than that of the SE module, because the ECA module reduces the complexity of the dependencies between channels through the cross-channel strategy, although it retains a certain degree of relationship between channels. However, this strategy does not achieve a good detection effect in the overall detection process. While the CBAM module separates the channel attention from the spatial attention, the overall detection effect is poor compared with the SE module and the ECA module.

The combination of attention mechanisms used in this paper is structurally similar to the CBAM module^36^, and both channels and coordinates are enhanced in sequence. CBAM uses CAM to focus on channels containing object information and SAM to localize objects on the image^37^. The method uses average pooling and maximum pooling operations to compress the feature map, and obtains the compression weight to adjust the weight of the feature map, so that the network pays more attention to the target features during the training process. The structure of the SE module used in this paper is similar to that of the CAM module, and both pay more attention to the channel where the target is located. This paper uses Coordinate Attention^38^ for spatial feature enhancement. Unlike SAM by adjusting the weight of space, this module can capture long-range dependencies along one spatial direction while preserving precise locations along the other direction. After encoding, a pair of direction-aware feature maps are applied to the output features. This method can save the precise position information in two directions in space , and can capture cross-channel information at the same time, so it achieves better detection results than CBAM.

The above groups of mainstream backbone networks are selected for comparison. The experimental results show that the network designed in this paper has achieved good detection results in both evaluation indicators. Compared with the second-place ResNet50 network, the detection network in this paper is improved by 3.7%, and compared with the worst, VGG16, by 26%, achieving a better detection effect. During the whole training process, the performance of our network has been better than other networks. As the training approaches closer to a value, our method shows better stability, indicating that the fusion attention mechanism strategy proposed in this paper is more robust for lung nodule detection.

We compared our method with state-of-the-art object detection methods in the field of computer vision, and the experimental results show that the method proposed in this paper has good performance.

### Summarize

In the problem of pulmonary nodule detection, due to the complex background of lung medical images, the easy interference of similar tissues, and the issue of the ease of submersion of small-sized pulmonary nodules, this paper designs a multi-scale pulmonary nodule based on the attention mechanism. In the detection network, pseudo-color processing is used in the preprocessing stage to introduce the contextual information of lung nodules. In the feature extraction part, the network uses the basic module ResSCBlock proposed in this paper, which introduces a compressed excitation unit and a coordinate attention unit to fuse the feature information and coordinate information of lung nodules, and introduces a feature pyramid for multi-scale lung nodules. Compared with the mainstream network, the detection network in this paper has better detection effect.

Since the proposed multi-scale lung nodule detection network based on attention mechanism lacks specialized detection schemes for different types of lung nodules, there is still room for improvement in the future. For example, a multi-stage detection network is designed to first judge the type of lung nodules. In addition, the network can only predict the location and size information of pulmonary nodules, but does not judge the characteristics of pulmonary nodules. These clinical manifestations also play an important role in judging the disease. Therefore, the proposed method in this paper needs to be further improved in the future.

## Data Availability

Medical lung imaging for training and validation is available from this website: https://drive.google.com/drive/folders/1q9EpTy_y8GRWnGJyRYa0aopyujmDm0Ye?usp=sharing, https://drive.google.com/file/d/1sdkbup0HkMp05isy_lzIKrl9Tkd-xWL1/view?usp=sharing ,and all the data generated during and/or analyzed during the current study are included in the manuscript.
